# Nutritionally Mediated Oxidative Stress and Inflammation

**DOI:** 10.1155/2013/610950

**Published:** 2013-06-13

**Authors:** Alexandra Muñoz, Max Costa

**Affiliations:** ^1^New York University School of Medicine, Nelson Institute of Environmental Medicine, 57 Old Forge Road, Tuxedo, NY 10987, USA; ^2^Department of Environmental Medicine, New York University, 57 Old Forge Road, Tuxedo, NY 10987, USA

## Abstract

There are many sources of nutritionally mediated oxidative stress that trigger inflammatory cascades along short and long time frames. These events are primarily mediated via NF**κ**B. On the short-term scale postprandial inflammation is characterized by an increase in circulating levels of IL-6 and TNF-**α** and is mirrored on the long-term by proinflammatory gene expression changes in the adipocytes and peripheral blood mononuclear cells (PBMCs) of obese individuals. Specifically the upregulation of *CCL2*/MCP-1, *CCL3*/MIP-1**α**, *CCL4*/MIP-1**β**, *CXCL2*/MIP-2**α**, and *CXCL3*/MIP-2**β** is noted because these changes have been observed in both adipocytes and PBMC of obese humans. In comparing numerous human intervention studies it is clear that pro-inflammatory and anti-inflammatory consumption choices mediate gene expression in humans adipocytes and peripheral blood mononuclear cells. Arachidonic acid and saturated fatty acids (SFAs) both demonstrate an ability to increase pro-inflammatory IL-8 along with numerous other inflammatory factors including IL-6, TNF**α**, IL-1**β**, and CXCL1 for arachidonic acid and IGB2 and CTSS for SFA. Antioxidant rich foods including olive oil, fruits, and vegetables all demonstrate an ability to lower levels of IL-6 in PBMCs. Thus, dietary choices play a complex role in the mediation of unavoidable oxidative stress and can serve to exacerbate or dampen the level of inflammation.

## 1. Introduction

**Figure 1:**
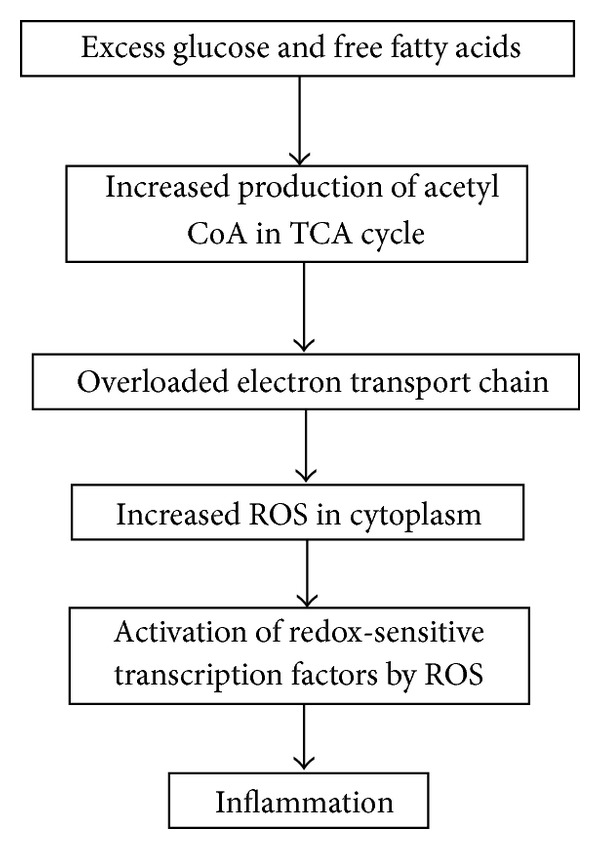
Downstream effects of nutrient overload. Overview of the downstream effects mediated by nutrient overload. Excess glucose and free fatty acids overwhelm the tricarboxylic acid (TCA) cycle which leads to an increase in the production of acetyl CoA. Excess acetyl CoA stimulates the mitochondria to produce excess superoxide in the electron transport chain, and the subsequent conversion of superoxide to hydrogen peroxide results in an increase of reactive oxygen species (ROS) within the cell. This change in redox status activates numerous redox-sensitive transcription factors, including NF*κ*B, which is the main mediator of inflammatory responses.

There are many sources of nutritionally mediated oxidative stress that trigger inflammation along short and long time frames. In order to focus the discussion of this topic this review will address how consumption of food, the quantity of food, and the macronutrient constituents serve as sources of oxidative stress and inflammation (Figure 1). On the short-term scale postprandial mitochondrial oxidative stress leads to inflammation, a process that is most strongly influenced by quantity and is mediated primarily by nuclear factor *κ*B (NF*κ*B), and on the long-term scale chronic overconsumption leads to obesity, which induces more permanent states of inflammation through the generation of white adipose tissue which secretes proinflammatory factors. Gene expression changes associated with obesity serve as a lens through which to view the short- and long-term consequences of nutritionally mediated oxidative stress and inflammation. There are additional mechanisms through which fats and glucose mediate inflammation, and these will be briefly discussed. Numerous human intervention studies have implemented various strategies to ameliorate the impact of nutritionally mediated inflammation changes in gene expression. Review of these studies highlights how pro-inflammatory and anti-inflammatory consumption choices mediate expression of a similar set of genes in post-prandial inflammatory states and in chronic inflammatory states in obese individuals and that such changes can be observed in both human adipocytes and peripheral blood mononuclear cells.

## 2. Oxidative Stress as a Diet-Induced Condition

### 2.1. From Nutrient Overload to Oxidative Stress to Inflammation

Overconsumption of food leads to dysmetabolism a state where energy intake exceeds energy expenditure, and cellular oxidative stress ensues [1, 2]. The increase in oxidative stress leads to numerous downstream effects including the induction of inflammatory cascades [1–3]. Figure 1 provides a simplified overview of the events that link overconsumption and inflammation. This process begins with mitochondrial overload of free fatty acids and glucose, which results in an increase in the production of acetyl coenzyme A (acetyl CoA), an enzyme important in cellular metabolism [4]. Higher levels of acetyl CoA result in an increase in reduced nicotinamide adenine dinucleotide (NADH) generation from the tricarboxylic acid (TCA) cycle. Increased availability of NADH increases electron generation by complex I of the mitochondrial electron transport chain and elevates membrane potential to the extent that complex III is stalled resulting in a longer half-life for coenzyme Q. Increased availability of coenzyme Q leads to an increased reduction of oxygen to superoxide (O_2_^∙−^). Thus the main impact of overconsumption of free fatty acids and glucose is higher levels of superoxide in the mitochondria [5]. Superoxide is a relatively unstable intermediate and in large part is converted to hydrogen peroxide in the mitochondria by superoxide dismutase. The newly formed hydrogen peroxide can then undergo a Haber-Weiss or Fenton reaction, yielding a highly reactive hydroxyl radical (^∙^HO), which can oxidize mitochondrial proteins, DNA, and lipids and amplify the effects of the superoxide-initiated oxidative stress [6, 7]. The generation of highly reactive oxygen radicals can activate redox-sensitive transcription factors and result in numerous downstream effects, including triggering inflammatory cascades and increasing ROS production. Questions remain regarding the permeability of the mitochondrial inner membrane to superoxide, and there is some evidence suggesting that superoxide can permeate anion channels, which may serve as an additional source of oxidative stress in the cytoplasm [8]. Depending on the cell types, the impact of this oxidative stress can result in various forms of dysfunction, making this a complex system to understand and track [9].

 It is important to note that there are additional mechanisms that can induce oxidative stress and inflammation both for glucose and free fatty acids, and those will be addressed in more detail later in this review. For now, the focus will remain on the oxidative stress induced by an overloaded TCA cycle. 

### 2.2. Consequences of Oxidative Stress

A strong theory has emerged in the literature that supports the idea that the excess generation of superoxide in the mitochondria and the subsequent generation of reactive oxygen species lead to the cell's inability to deal with chronic mitochondrial oxidative stress and that the consequences of such stress in various cell types are responsible for several conditions including cardiovascular disease, type 2 diabetes mellitus, obesity, and metabolic syndrome. The sequelae of events that lead to these conditions have been detailed in the comprehensive review provided by Ceriello and Motz [5]. In short, the oxidative stress impacts pancreatic *β* cells by leading to the decreased expression of glucose transporter type 4 (GLUT4), which over time can support the onset of type 2 diabetes mellitus. In endothelial cells the oxidative stress primarily impacts cell function through peroxynitrite formation, a highly favorable reaction that reduces nitric oxide (NO) availability, resulting in defective endothelial dependent vasodilation, which on the long-term scale leads to cardiovascular disease [10]. 

Nutritionally mediated oxidative stress may also play a role in cancer development. Oxidative stress can alter the epigenetic program by interacting with the activity of the dioxygenase family of enzymes and in turn can lead to changes in histone methylation which alters gene expression [11]. Epigenetic changes induced by oxidative stress can promote the progression of gene expression changes that have been associated with the progression of cancer [12]. 

Overloading of the TCA cycle may also result in additional epigenetic responses due to fluctuations in the steady-state dynamics of cellular metabolism and the reliance of histone modifying enzymes on acetyl CoA. Histone acetyltransferases (HATs) utilize the acetyl group in acetyl CoA to acetylate the lysine residue on the N-terminal of histones, which serves to promote a more open chromatin formation and increased gene expression, while histone deacetylases (HDACs) reverse this process and promote a more closed chromatin formation that reduces gene expression [13]. HATs and HDACs rapidly cycle in applying and removing acetyl groups, and it was recently demonstrated that the regulation of HATs and HDACs occurs in response to changes in the steady-state dynamics of metabolic products and is responsive to intracellular pH [14]. *In vitro* assessments have demonstrated that coenzyme A (CoA) derivatives, including acetyl CoA, butyryl CoA, malonyl CoA, and NADPH stimulate class I HDACs on histones, while free CoA inhibits HDAC activity [15]. Thus changes in the steady-state of acetyl CoA brought on by an overloaded TCA cycle may also impact histone acetylation dynamics and lead to changes in histone acetylation and gene expression. 

 Oxidative stress can also serve to promote cancer by influencing telomere length. A study conducted on men from the Framingham study found that oxidative stress and insulin resistance are inversely associated with telomere length [16]. Telomere length then reflects the lifelong burden of oxidative stress and its cumulative impact on insulin resistance. Because long telomeres are an important barrier against aberrant segregation events in mitosis, which protects the cell from aneuploidy, a hallmark of cancer cells [17], this finding further underscores the importance of minimizing oxidative stress generated by mitochondrial overload to protect against cancer.

## 3. Obesity and Inflammation

### 3.1. From Oxidative Stress to Inflammation

 The food-induced increase in oxidative stress also corresponds to an increase in inflammation (Figure 1), and this increase in inflammation can be observed through alterations in numerous signaling pathways and immune system processes. Oxidative stress can modulate numerous redox-sensitive transcription factors including NF*κ*B, activator protein 1 (AP-1), and early growth response 1 (EGR1), which can collectively engage cellular and systemic inflammation in a strong feed-forward process [18, 19]. The NF*κ*B mediated release of inflammatory cytokines (tumor necrosis factor alpha (TFN*α*) and interleukin-6 (IL-6)), and acute phase reactants (C-reactive protein (CRP)) are the most commonly addressed pathways linking food consumption and inflammation in human studies. 

On longer-time scales excess free fatty acids are stored as triglycerides in adipocytes. Brown adipocytes primarily serve to promote thermogenesis and play a major role in the formation of “baby fat,” while white adipocytes regulate endocrine function with the secretion of the hormone leptin [20]. In the onset of obesity the accumulation of white adipose tissue generates an additional set of factors that contribute to inflammatory cascades. For instance, adipose tissue often exhibits hypoxia which leads to induction of hypoxia inducible factor 1 alpha (HIF-1*α*) and the expression of inflammation-related adipokine genes including leptin, vascular endothelial growth factor (VEGF), and angiopoietin-like protein 4 (ANGPTL4) that serve to perpetuate the state of inflammation [21]. Changes in the white adipose tissue promote local and systemic inflammation and will be discussed in more detail in “Obesity and Inflammation” below.

### 3.2. Postprandial Inflammation

On a short-term scale the consumption of food leads to certain levels of oxidative stress and inflammation after every meal as discussed via overloaded mitochondrial metabolism (Figure 1). Studies that assess postprandial gene expression support the idea that food consumption increases inflammation and have determined that the level of inflammation can be impacted by the amount of calories consumed at a sitting, as well as the glycemic index and the fatty acid profile of the meal [22]. Postprandial inflammation is triggered by blood glucose levels, which act on inflammatory processes in a dose-dependent manner such that meals with higher glycemic index induce increased inflammatory response relative to meals with lower glycemic index [23]. Thus the magnitude of the blood glucose peak is not only strongly influenced by the macronutrient composition of the meal, but it is also influenced by the amount of the food consumed such that a well-balanced meal may still cause a substantial peak if the serving size is excessive [2]. Human intervention studies assessing postprandial inflammation have found that reducing the glycemic index [23, 24] of a meal and caloric restriction [25, 26] result in downregulation of immunological genes and their inflammatory processes.

### 3.3. Obesity and Inflammation

Macronutrient consumption and habitual overconsumption of food have the consequence of producing chronic levels of inflammation and the upregulation of adhesion molecules, leading to infiltration of the adipose tissue with macrophages. Over time the accumulation of macrophages and monocytes in the tissue alter the nature of the tissue, and the extensive tissue remodeling turns the adipose tissue into an endocrine organ that can mediate further levels of inflammation [18]. Adipocytes found in white adipose tissue exhibit altered physiology due to excess fat storage and release numerous pro-inflammatory cytokines and chemokines including TNF-*α*, IL-6, leptin, resistin, visfatin, adiponectin, monocyte chemotactic protein-1 (MCP-1), and plasminogen activator inhibitor-1 (PAI-1), which serve to recruit additional immune cells and promote infiltration of macrophages, leading to a strong inflammatory cycle and eventually to insulin resistance at local and systemic levels [3, 18, 27]. Circulating levels of IL-6 and TNF*α* are strongly correlated with increasing adipose mass [28]. There is also evidence to support the idea that peripheral blood mononuclear cells (PBMCs) may also mediate the increase of the pro-inflammatory cytokines in obese states [29]. 

Later studies in this area have confirmed the pro-inflammatory state and further characterized the monocyte-macrophage system, where two types of macrophages mediate the inflammatory profile. In obese subjects pro-inflammatory macrophages (M1) predominate over anti-inflammatory macrophages (M2) [30, 31]. The M2 macrophages are alternatively activated by interleukin-4 (IL-4) stimulation and the peroxisome proliferator-activated receptor gamma (PPAR*γ*) receptor and have been demonstrated to protect against the metabolic consequences of obesity in mice [32]. In humans, there is evidence to suggest that (PPAR*γ*) upregulation coincides with increased expression of interleukin-10 (IL-10), an anti-inflammatory cytokine and M2 marker, suggesting that IL-10 expression and M2 dominance are correlated [33]. Expression of IL-10 appears to be complex, such that individuals exhibiting symptoms of metabolic syndrome, whether obese or nonobese, exhibit lower levels of IL-10 compared to their obese and nonobese counterparts [34], possibly due to the distribution of M1/M2 macrophages. Moreover, levels of IL-10 in nonobese but overweight female adolescents have been correlated with levels of TNF*α* and IL-6 suggesting that, in more healthy but still overweight phenotypes, IL-10 is upregulated to suppress inflammation [35]. 

### 3.4. Obesity-Linked Changes in Gene Expression

Obesity-linked changes in gene expression are important to note as they are strong markers for the long-term consequences of nutritionally mediated inflammation. In large part these changes are likely mediated by the hormone leptin, which is released by the adipose tissue and plays various complex roles in the body including acting as an immunomodulating and pro-inflammatory agent [36]. 

Changes in gene expression resulting from obesity-linked inflammation are observed in both adipocytes and in peripheral blood mononuclear cells. In a microarray study that compared the gene expression profile of adipocytes of obese and nonobese Pima Indians, the major changes in gene expression profiles were observed in relation to inflammation related genes. The majority of the differentially expressed inflammation related genes (52/54) were upregulated in the adipocytes including chemokines monocyte chemoattractant protein-1 (MCP-1/CCL2), macrophage inflammatory protein (MIP-1*α*/CCL3), MIP-1*β*/CCL4, chemokine (C-X-C motif ligand 1 (CXCL1), macrophage inflammatory protein 2*α* (MIP-2*α*/CXCL2), MIP-2*β*/CXCL3, and stromal cell-derived factor 1 (SDF-1/CXCL12) [37]. Elevated levels of MCP-1 and MIP-1*α* serve to attract monocytes and macrophages to adipose tissue, and their presence is supported by numerous studies, which indicate that the percentage of adipose tissue comprised of macrophages is correlated with obesity [27]. TNF*α* was excluded from the list of differentially expressed genes because it did not pass with FDR correction, though phosphatidylinositide 3-kinase (PI3K), a member of a downstream pathway associated with TNF*α*, was significantly overly represented in gene ontology (GO) terms. There was also an upregulation of interferon-induced genes.

A pro-inflammatory state has also been observed in the PBMCs of obese individuals. This state is characterized by an increase in NF*κ*B binding activity in the nucleus and p65 expression, as well as a decrease in I kappa B kinase subunit b (IKKB-B) in the mononuclear cells. Additionally, NF*κ*B regulated genes also exhibit up-regulation in this state and include *TNF*α**, *IL-6*, migration inhibitory factor* (MIF)*, and matrix metallopeptidase 9* (MMP-9)* [29]. 

The use of microarray studies in this area is still being established, and there is debate in the field as to whether it is more appropriate to assess changes via gene expression patterns found in subcutaneous adipose tissue or in peripheral blood mononuclear cells. The ease of collection for PBMCs is favorable for study implementation, but the extent to which patterns are consistent between adipose tissue and PBMCs requires additional study. One study which evaluated the expression of inflammatory cytokines associated with truncal fat found a strong correlation between the level of truncal fat and the mRNA levels in PBMCs of various inflammatory markers [38]. 

## 4. Lipid and Glucose Specific Pathways to Inflammation

### 4.1. Ω-6 Fatty Acids

Fatty acids and their derivatives eicosanoids can serve as signaling molecules that interact with numerous transcription factors to promote downstream effects. Peroxisome proliferator-activated receptors (PPARs) are ligand-activated transcription factors that serve as sensors of lipid levels. Fatty acids and various fatty acid derived compounds can serve as ligands, and among them PPARs demonstrate a general preference for long-chain polyunsaturated fatty acids (PUFAs) [39, 40]. Dietary PUFAs also interact with sterol regulatory element binding protein (SREBP), and their transcription in the liver is involved in the regulation of genes related to synthesis and uptake of cholesterol, fatty acids, and phospholipids, and in addition SREBPs are implicated as early mediators of insulin responses [41]. *NF-E2 related factor-2 (NRF2)*, which serves widely as an oxidative stress response factor, exhibits up-regulation in response to the oxidized products of eicosapentaenoic acid ((EPA) 20 : 5 *ω*-3) and docosahexaenoic acid ((DHA) 22 : 6 *ω*-3), thereby mediating oxidative stress responses and providing experimental support to the idea that the oxidative quality of fat supplements requires careful regulation [40, 42]. 

Arachidonic acid ((AA) 20 : 4n-6) is a PUFA that has substantial evidence supporting its role in pro-inflammatory conditions. Arachidonic acid is widely available for intake and can be found in high quantities in many food items including fish, white meat, red meat, eggs, and dairy and also in vegetable oils such as peanut oil, canola oil, and sesame oil. The term “Western diet” is typically used to describe the modern diet that is a product of the industrial and agricultural revolutions. Western diets are characterized by consumption of an increased proportion of fat and refined sugar, reduced proportion of complex carbohydrate and fiber intake, and reduced proportion of fruit and vegetable consumption [43]. The proportions of consumption in the Western diet are often highlighted as a contrast from more traditional diets, such as the Okinawan diet, which includes higher consumption of complex carbohydrates, fiber, fruits, and vegetables and lower consumption of animal products and their fats [44]. Therefore in Western diets AA is a major source of PUFA as it is found in eggs, dairy, fish, and meats.

AA is a key player in promoting inflammation, because it is the precursor for numerous eicosanoids, which are fatty acid derived molecules that mediate inflammatory responses [45]. Eicosanoids which include prostaglandins (PGs), thromboxanes, and leukotrienes are derived from 20 carbon PUFAs, and inflammatory cells are dominated by the presence of *ω*-6 20 carbon PUFAs making AA metabolism central to inflammation and pharmacological approaches aimed at reducing inflammation. AA is converted by the enzymes cyclooxygenase- (COX-) 1 and COX-2, the inducible form, to PGs of which prostaglandin E2 (PGE_2_) is primarily known for its pro-inflammatory effects, and also has less well-known anti-inflammatory effects such as its ability to inhibit the pro-inflammatory cytokines TNF-*α* and interleukin-1 (IL-1) which were demonstrated in *in vitro* [46]. The evidence supporting AA's role as a key player in inflammation and disease however is strong, and COX-2 up-regulation occurs during NF*κ*B activation and in response to IL-1B [47, 48]. *In vitro* studies utilizing human prostate cancer cells have found that AA induces COX-2, which is significant because prostate cancer and colorectal cancers consistently exhibit increased levels of COX-2 and PGE_2_ [49, 50]. In addition, AA has been shown to induce 11 genes regulated by NF*κ*B in a human prostate cancer cell line PC-3 including *COX-2*, *I*κ*Ba*, *NF*κ*B*, granulocyte macrophage stimulating factor* (GM-CSF)*, *IL-1B*, *CXCL-1*, *TNF-*α**, *IL-6*, *LTA*, *IL-8*, *PPAR*γ**, *PPAR*δ**, and intercellular adhesion molecule 1* (ICAM-1)*. AA's effects begin as early as five minutes when added *in vitro *to prostate cancer cells at which time PI3K exhibits significant activation with activation of Akt and nuclear translocation of NF*κ*B following at 30 minutes [51]. 

### 4.2. Saturated Fatty Acids

While there is substantial evidence to suggest that saturated fatty acids (SFAs) can induce pro-inflammatory signaling, the interactions of saturated fatty acids are still rather ambiguous in many areas, and care needs to be taken when addressing their effects as the lengths of saturated fatty acid chains can produce varying physiological effects and many mechanisms are still debated [52]. Long-chain saturated fatty acids are typically cited for their harmful effects to endothelial cells and include acids such as myristate and palmitate which can induce apoptosis via NF*κ*B induction in human coronary artery endothelial cells (HCAECs) [53]. Further studies in this area have indicated that long-chain SFA can induce pro-inflammatory endothelial cell phenotypes via incorporation into endothelial cell lipids and that short- and medium-chain SFAs do not incorporate or cause lipotoxicity. Specifically, stearic acid induced an up-regulation of *ICAM-1* human aortic endothelial cells (HAECs) in an NF*κ*B dependent manner [54]. 

One area of contention surrounds the question of whether SFAs mediate NF*κ*B inflammatory effects and the induction of COX-2 through the Toll-like receptors (TLRs). Controversy in this area arises from technical issues of contamination that may occur from endotoxins which are capable of activating TLRs. Recent studies in this area have taken care to purify reagents and, despite these precautions, have still produced conflicting reports. In one study investigators found that SFA (lauric acid and palmitic acid) did not activate TLR2 and TLR4 [55] in HEK-Blue cells transfected with TLR2 and TLR4, but in another study investigators found that SFA (lauric acid and palmitic acid) did activate TLR2 and TLR4 in RAW264.7 macrophages and transiently transfected THP-1 monocytes [56]. 

Human studies assessing the impact of SFA on gene expression are limited, but there are numerous epidemiologic studies, which assess the relationship between SFA intake and cardiovascular disease, an inflammatory condition. Meta-analyses of prospective studies assessing the association between cardiovascular disease and saturated fat found a consistent lack of an association, and metaregressions performed on randomized trials that substituted PUFA for SFA found there was no change in risk for cardiovascular disease with the fat substitution [57]. Lack of conclusiveness in these studies may result from the fact that SFAs are generally grouped together, and medium-chain SFAs have been shown to provide beneficial health effects including suppression of body fat accumulation and obesity [58, 59]. One human study which aimed to assess the impact of a SFA diet versus a monounsaturated fatty acids (MUFAs) diet on gene expression in adipose tissue found that the SFA diet led to an overexpression of genes involved in inflammatory processes. They found that the gene expression profile included upregulation of cathepsin S* (CTSS)* interleukin-8 *(IL-8)*, integrin beta 2 *(ITGB2) *in moderately overweight individuals and that the profile was similar to that found in obese Pima Indians, concluding that changes were associated with diet-induced changes rather than due to obesity [60]*.*

### 4.3. Glucose

Postprandial hyperglycemia provides another series of mechanisms through which consumption of food can induce inflammatory cascades and which over time can lead to the inflammation related condition of type 2 diabetes [61]. The results of these glucose excursions are mediated via an increase in oxidative stress likely initiated by the same mitochondrial overload previously discussed, but glucose provides an additional set of mechanisms with which the oxidative stress and associated inflammation can manifest. For instance, oxidative stress in the presence of intracellular hyperglycemia results in the production of reactive intracellular dicarbonyls which react with amino acids to form advanced glycation end (AGE) products that go on to bind AGE receptors and induce expression of inflammatory cytokines in macrophages and procoagulatory and proinflammatory molecules in endothelial cells [62]. 

Acute hyperglycemia results in elevated levels of circulating inflammatory cytokines including TNF*α*, IL-6, and IL-18 and more extreme responses in these parameters are observed when glucose spikes; this response is attenuated by administration of glutathione confirming the presence of an oxidative stress-related mechanism [1]. Individuals with diabetes are particularly susceptible to postprandial glucose spikes and these peaks spike oxidative stress to a greater degree than sustained hyperglycemia [4]. Additionally, even in normal subjects hyperglycemia induces an increase in circulating levels of serum-soluble intercellular adhesion molecule-1 (sICAM-1) indicating that glucose excursions can initiate atherogenic events in nondiabetic individuals [63].

## 5. Nutritional Strategies to Ameliorate Inflammation

While it is evident that high levels of consumption of macronutrients can increase oxidative stress and produce inflammation through NF*κ*B mediated pathways, as well as via alternative mechanisms, such as through excessive *ω*-6 stimulated inflammation, there are other dietary choices that can simultaneously reduce inflammation. Much information about these dietary choices comes from epidemiologic evidence, which indicates that the Mediterranean and Okinawan diets of the Greek and Japanese populations, respectively, are associated with significantly lower levels of type 2 diabetes, cardiovascular disease, metabolic syndrome, and cancer [44, 64]. The Okinawan diet, in particular, is marked by consumption of minimally processed foods that are rich in antioxidants, have low glycemic index, and are supported culturally by smaller portion sizes. The Okinawan diet is rich in vegetables, low glycemic index beans, and sweet potatoes and contains small amounts of fish and lean meats [44, 65]. Each of these diets is also rich in virgin olive oil or fish oil, which play critical roles in dampening inflammation and will be discussed in detail here. Thus these diets promote less inflammation due to consumption of smaller meals comprised of minimally processed foods (e.g., vegetables and legumes) and include foods that dampen inflammation, such as healthful fats and antioxidants. 

### 5.1. Caloric Restriction and Macronutrient Balance

The extent to which macronutrient composition and caloric restriction independently affect gene expression patterns is unclear as most studies implement both strategies in their interventions. One study that independently assessed the impact of a macronutrient balanced diet found that the intervention diet (30 : 30 : 40 energy percent from carbohydrates, proteins, and fat, resp.) which had higher protein and less fat than the prestudy diet (41 : 19 : 40) yielded immediate and persistent downregulation in immunological genes in PBMCs [66]. Attempts to sort out the key signal have been made, and one study which assessed the impact of both factors in gene expression of adipose tissue in obese women found that caloric restriction had a more profound impact on adipose tissue gene expression than macronutrient composition [67]. The effects of caloric restriction on inflammatory profiles have been well documented but typically take time to shift the profile, likely due to its impact on weight loss and the adipocyte generation of inflammation. Obese women undergoing intense caloric restriction for 28 days exhibited an improvement in the inflammatory profile of 100 transcripts in subcutaneous adipose tissue, including downregulation of inflammatory markers including acute phase reactants and TNF-related proteins, as well as a simultaneous upregulation of anti-inflammatory markers such as IL-10 and IL-1. These changes were only observed after a 28-day period and not after 2 days [25]. Similarly, gene expression evaluation by microarray in PBMCs demonstrated that caloric restriction downregulates genes involving oxidative phosphorylation such as NDUFS2 (NADH-coenzyme Q reductase) and inflammatory cytokines, including IL-8 [26]. In another study that evaluated the long-term effects of caloric restriction, which was implemented via gastric bypass surgery, microarray analysis of gene expression in adipose tissue also indicated a significant down-regulation of numerous inflammatory markers including *IL-6*, *IL-8*, *IL-1B*, *CCL2*/MCP1, *HIF1*α**, and *PTGS2*/COX-2. In addition there was a significant up-regulation of homeobox transcription factors (*HOXA5*, *HOXA9*, *HOXB5*, and *HOXC6*) that may be involved in a metabolically favorable remodeling of adipose tissue after fat loss, however, because downstream targets of homeobox genes have not yet been identified their exact role in this process or relationship to fat loss remains unknown [68]. 

### 5.2. Fish Oil

Evolutionary evidence suggests that humans evolved eating a diet where the ratio of *ω*-6 to *ω*-3 was approximately 1, and over the last 50 years the ratio has increased from 2 : 1 to 25 : 1, and thus the idea that greater incorporation of *ω*-3 into the diet is important for health has gained general acceptance [69]. However it is specifically the *ω*-3s found in fish oil, DHA and EPA, that are implicated in improved health through numerous epidemiologic studies. A hallmark study in the field of *ω*-3 fatty acids was the 1970s epidemiologic study, which found that Inuit consumed more than 14 g per day of *ω*-3 fatty acids and that their rate of myocardial infarction was 10 times lower than the rate among Danes who consumed only 3 g per day [70]. Many studies have investigated this relationship, and there is substantial evidence in support of it, though there are some conflicting reports [71]. The complexity of this issue may be part of the cause of such confusion because the key factors in understanding the role of *ω*-3s in the diet have yet to be fully elucidated. The concept that is mostly unclear is whether it is the total ratio of *ω*-6 to *ω*-3 PUFA, the ratio of long-chain *ω*-6 to *ω*-3 PUFA, or the presence of high concentrations of *ω*-3 PUFA that is the key factor [72]. What has been made clear through studies focused on cardiovascular disease, as well as other inflammatory conditions, is that, unlike Inuit diets that are rich in *ω*-3, Western diets are typically rich in *ω*-6 PUFA and exhibit ratios of *ω*-6 to *ω*-3 that are well beyond recommended ratios. Associated with these ratios and low levels of *ω*-3 are a host of diseases including autoimmune diseases, allergies, asthma, and cancer [69]. 

Numerous human studies have observed that *ω*-3 PUFAs found in fish oil have the capacity to produce therapeutic effects on a number of diseases including cardiovascular disease [71, 73] and rheumatoid arthritis [74]. Increased consumption of *ω*-3 fatty acids is associated with anti-inflammatory effects that result from reduced AA-derived eicosanoids due to competitive inhibition for enzymes, reduced triglyceride levels, and inhibition of platelet aggregation [71]. In a human intervention study, healthy individuals were placed on diets that controlled for caloric intake, as well as fat intake, for 1 week and then were provided with supplements of fish oil containing long-chain EPA and DHA and borage oil containing short-chain gamma linolenic acid (GLA) for 4 weeks. There was an observed decrease in the levels of PI3K*α* and -*γ* but not in its downstream effectors AKT/NF*κ*B. PI3K*δ* and PI3k*γ* are thought to be involved in the inflammatory response [75, 76].

The relationship between fish oil supplementation and fluctuations in IL-10 is another interesting point of interaction. In one human study supplementation with a combination of fish oils and borage oil significantly decreased expression for *IL-1B*, *IL-10*, and *IL-23*. *IL-5 *and *IL-17* exhibited strong but not significant down-regulation as well. No effect was observed on a number of enzymes involved in leukotriene production suggesting that the observed changes were caused by substrate availability [72]. In another human study involving obese patients, supplementation with EPA increased *IL-10* levels [33]. The discrepancy between the direction of change for the *IL-10 *expression in these studies may be the result of supplementation in obese versus normal weight patients, suggesting that there are nuanced regulatory mechanisms in place. A nuanced regulation may mediate responses to EPA supplementation in relation to the given phenotype (obese or normal). Such a nuanced response would support the underlying logic that EPA may shift macrophage dominance in obese patients from the more pathologic M2 state to a more healthful M1 state in which IL-10 is initially upregulated in the M2 state to suppress excess inflammation and then downregulated when the M1 state is achieved. 

### 5.3. Extra Virgin Olive Oil

The Mediterranean diet has been associated with lower incidence of cardiovascular events, obesity, diabetes, and cancer [64, 77, 78]. One of the key components of a Mediterranean diet is high consumption of extra virgin olive oil (VOO), which can be rich in oleic acid and phenolic compounds that contain antioxidant and anti-inflammatory capabilities. In a post-prandial state consumption of VOO has been shown to reduce inflammatory markers and improve levels of antioxidants in serum [79]. Other studies have demonstrated that in a postprandial state VOO can reduce the NF*κ*B inflammatory response in PBMCs compared to diets enriched with fat from butter and walnuts [80]. In a microarray study assessing the impact of acute early morning VOO consumption in obese individuals, VOO was found to downregulate genes in the NF*κ*B pathway and specifically down-regulate the expression of multiple inflammatory genes including *PTGS2, IL1B, CCL3, CXCL1, CXCL2, CXCL3, CXCR4, IL-6,* and oncostatin M* (OSM)* [81]. 

### 5.4. Dietary Antioxidants and Phytonutrients

Another intervention that can attenuate diet-induced oxidative stress is the inclusion of dietary antioxidants and phytonutrients, which dampen down the oxidant stress that is generated during metabolism of glucose or fatty acids in the TCA cycle during any meal. Deeply pigmented foods such as berries, red wine, dark chocolate, tea, and pomegranates are rich sources of antioxidants that are shown to mitigate the effects of oxidant production and protect the vascular endothelium [2]. Evidence from a human study indicates that oxidative stress of a high-fat meal can be mitigated by coconsumption of dietary antioxidants with the high-fat meal [82]. The inclusion of cinnamon in a glucose-rich meal delays gastric emptying and significantly reduces the postprandial glucose excursion which aids in dampening inflammation [83, 84] but does not have this effect following a high-fat meal [85]. 

The phytonutrients and antioxidants found in fruits and vegetables have been demonstrated in human studies to impact inflammatory markers in the PBMCs of young adults. After adjustment for possible confounding factors including age and fiber intake, the highest tertile of fruit and vegetable consumption was found to be associated with the lowest levels of CRP, homocysteine, *ICAM1*, interleukin receptor 1 (*ILR1*), *IL6, TNF*α**, and *NF*κ*B* gene expression in young adults [86]. 

## 6. Conclusion

 The consumption of food and the subsequent cellular metabolism of fatty acids and glucose produce, even under normal circumstances, oxidative stress which triggers an NF*κ*B mediated response that invokes inflammatory factors. Post-prandial inflammation is characterized by an increase in IL-6 and TNF-*α* in both normal individuals and those with diabetes. Obese individuals have chronically elevated levels of IL-6 and TNF*α*, and their adipose tissue and PBMCs exhibit increased expression of inflammatory genes. Specifically, *CCL2*/MCP-1, *CCL3*/MIP1*α*, *CCL4*/MIP-1*β*, *CXCL2*/MIP-2*α*, and *CXCL3*/MIP-2*β* are noted because these have been observed to be elevated in both adipocytes and PBMCs of obese humans. 

AA and SFA both demonstrate an ability to increase *IL-8* along with numerous other inflammatory factors including *IL-6*, *TNF*α**, *IL-1*β*,* and *CXCL1* for *ω*-6 AA, and *IGB2* and *CTSS *for SFA. Dietary strategies aimed at reducing chronic levels of inflammation prove effective and are centered around caloric restriction and inclusion of foods, which either dampen oxidative stress through the use of antioxidants and/or mediate anti-inflammatory signaling. Caloric restriction demonstrates an ability to reduce the level of the proinflammatory *IL-8* in PBMCs and is most effective when weight loss ensues. Notably antioxidant rich foods including olive oil, fruits, and vegetables all demonstrate an ability to lower levels of *IL-6* in PBMCs. 

Thus, dietary choices play a complex role in the mediation of unavoidable oxidative stress, and certain choices can either exacerbate or dampen that process as downstream interactions lead to transcription of pro- or anti-inflammatory factors. Moreover, the cumulative impact of long-term oxidative stress that leads to inflammatory conditions such as obesity are increasingly recognized as central factors in the development of cancer. While the full spectrum of mechanisms which link cancer and obesity is not fully elucidated and may include emerging factors such as microbiome composition [87], there is strong epidemiological evidence to support the risk of several types of cancer, such as colon, breast, endometrium, liver, kidney, gastric, gallbladder, and others with obesity, and mechanistic evidence to support the role of inflammation in this process [88]. 

## Abbreviations

acetyl CoA: Acetyl coenzyme A
AP-1: Activator protein 1
AGE: Advanced glycation end
ANGPTL4: Angiopoietin-like protein 4
AA: Arachidonic acid
CTSS: Cathepsin S
CRP: C-reactive protein
COX: Cyclooxygenase
DHA: Docosahexaenoic acid
EGR1: Early growth response 1
EPA: Eicosapentaenoic acid
(GLA): Gamma linolenic acid
GAPDH: Glyceraldehyde 3-phosphate dehydrogenase
GLUT4: Glucose transporter type 4
GM-CSF: Granulocyte macrophage stimulating factor
HATs: Histone acetyltransferases
HDACs: Histone deacetylases
HIF-1*α*: Hypoxia inducible factor 1 alpha
IKKB-B: I kappa B kinase subunit b
iNOS: Inducible nitric oxide synthase
*ITGB2*: Integrin beta 2
ICAM-1: Intercellular adhesion molecule 1
IL: Interleukin
MIP: Macrophage inflammatory protein
MMP-9: Matrix metallopeptidase 9
MIF: Migration inhibitory factor
MCP-1: Monocyte chemotactic protein-1
MUFAs: Monounsaturated fattyacids
NADH: Nicotinamide adenine dinucleotide
NO: Nitric oxide
NF*κ*B: Nuclear factor*κ*B
NRF2: Nuclear factor-E2 related factor-2
PPAR: peroxisome proliferator-activated receptor
PI3K: Phosphatidylinositide 3-kinase
PAI-1: Plasminogen activator inhibitor-1
PARP: Poly-ADP ribose; polymerase
PUFA: Polyunsaturated fatty acid
PGE_2_: Prostaglandin E2
PKC: Protein kinase C
SFAs: Saturated fatty acids
sICAM-1: Serum-soluble intercellular adhesion molecule-1
SREBP: Sterol regulatory element binding protein
SDF: Stromal cell-derived factor
TLRs: Toll-like receptors
TFN-*α*: Tumor necrosis factor alpha
TCA: Tricarboxylic acid
VEGF: Vascular endothelial growth factor
VOO: Virgin olive oil.

## References

[1] Esposito K, Nappo F, Marfella R (2002). Inflammatory cytokine concentrations are acutely increased by hyperglycemia in humans: role of oxidative stress. *Circulation*.

[2] O’Keefe JH, Gheewala NM, O’Keefe JO (2008). Dietary strategies for improving post-prandial glucose, lipids, inflammation, and cardiovascular health. *Journal of the American College of Cardiology*.

[3] van Gaal LF, Mertens IL, de Block CE (2006). Mechanisms linking obesity with cardiovascular disease. *Nature*.

[4] Monnier L, Mas E, Ginet C (2006). Activation of oxidative stress by acute glucose fluctuations compared with sustained chronic hyperglycemia in patients with type 2 diabetes. *Journal of the American Medical Association*.

[5] Ceriello A, Motz E (2004). Is oxidative stress the pathogenic mechanism underlying insulin resistance, diabetes, and cardiovascular disease? The common soil hypothesis revisited. *Arteriosclerosis, Thrombosis, and Vascular Biology*.

[6] Ott M, Gogvadze V, Orrenius S, Zhivotovsky B (2007). Mitochondria, oxidative stress and cell death. *Apoptosis*.

[7] Yin F, Sancheti H, Cadenas E (2012). Mitochondrial thiols in the regulation of cell death pathways. *Antioxidants & Redox Signaling*.

[8] Andreyev AY, Kushnareva YE, Starkov AA (2005). Mitochondrial metabolism of reactive oxygen species. *Biochemistry*.

[9] Hotamisligil GS (2006). Inflammation and metabolic disorders. *Nature*.

[10] Wright E, Scism-Bacon JL, Glass LC (2006). Oxidative stress in type 2 diabetes: the role of fasting and postprandial glycaemia. *International Journal of Clinical Practice*.

[11] Chervona MCY (2012). Histone modifications and cancer: biomarkers of prognosis?. *American Journal of Cancer Research*.

[12] Kurdistani SK (2010). Histone modifications in cancer biology and prognosis. *Epigenetics and Disease*.

[13] Yang X-J, Seto E (2007). HATs and HDACs: from structure, function and regulation to novel strategies for therapy and prevention. *Oncogene*.

[14] McBrian MA, Behbahan IS, Ferrari R (2013). Histone acetylation regulates intracellular pH. *Molecular Cell*.

[15] Vogelauer M, Krall AS, McBrian MA, Li J-Y, Kurdistani SK (2012). Stimulation of histone deacetylase activity by metabolites of intermediary metabolism. *Journal of Biological Chemistry*.

[16] Demissie S, Levy D, Benjamin EJ (2006). Insulin resistance, oxidative stress, hypertension, and leukocyte telomere length in men from the Framingham Heart Study. *Aging Cell*.

[17] Rajagopalan H, Lengauer C (2004). Aneuploidy and cancer. *Nature*.

[18] Iyer A, Fairlie DP, Prins JB, Hammock BD, Brown L (2010). Inflammatory lipid mediators in adipocyte function and obesity. *Nature Reviews Endocrinology*.

[19] Shoelson SE, Herrero L, Naaz A (2007). Obesity, inflammation, and insulin resistance. *Gastroenterology*.

[20] Henry SL, Bensley JG, Wood-Bradley RJ, Cullen-McEwen LA, Bertram JF, Armitage JA (2012). White adipocytes: more than just fat depots. *The International Journal of Biochemistry and Cell Biology*.

[21] Goossens GH (2008). The role of adipose tissue dysfunction in the pathogenesis of obesity-related insulin resistance. *Physiology and Behavior*.

[22] Margioris AN (2009). Fatty acids and postprandial inflammation. *Current Opinion in Clinical Nutrition and Metabolic Care*.

[23] Dickinson S, Hancock DP, Petocz P, Ceriello A, Brand-Miller J (2008). High-glycemic index carbohydrate increases nuclear factor-*κ*B activation in mononuclear cells of young, lean healthy subjects1-3. *American Journal of Clinical Nutrition*.

[24] Kallio P, Kolehmainen M, Laaksonen DE (2007). Dietary carbohydrate modification induces alterations in gene expression in abdominal subcutaneous adipose tissue in persons with the metabolic syndrome: the FUNGENUT Study. *American Journal of Clinical Nutrition*.

[25] Clément K, Viguerie N, Poitou C (2004). Weight loss regulates inflammation-related genes in white adipose tissue of obese subjects. *FASEB Journal*.

[26] Crujeiras AB, Parra D, Milagro FI (2008). Differential expression of oxidative stress and inflammation related genes in peripheral blood mononuclear cells in response to a low-calorie diet: a nutrigenomics study. *OMICS*.

[27] Weisberg SP, McCann D, Desai M, Rosenbaum M, Leibel RL, Ferrante AW (2003). Obesity is associated with macrophage accumulation in adipose tissue. *Journal of Clinical Investigation*.

[28] Bastard J-P, Jardel C, Bruckert E (2000). Elevated levels of interleukin 6 are reduced in serum and subcutaneous adipose tissue of obese women after weight loss. *Journal of Clinical Endocrinology and Metabolism*.

[29] Ghanim H, Aljada A, Hofmeyer D, Syed T, Mohanty P, Dandona P (2004). Circulating mononuclear cells in the obese are in a proinflammatory state. *Circulation*.

[30] Lumeng CN, Bodzin JL, Saltiel AR (2007). Obesity induces a phenotypic switch in adipose tissue macrophage polarization. *Journal of Clinical Investigation*.

[31] Lumeng CN, Delproposto JB, Westcott DJ, Saltiel AR (2008). Phenotypic switching of adipose tissue macrophages with obesity is generated by spatiotemporal differences in macrophage subtypes. *Diabetes*.

[32] Odegaard JI, Ricardo-Gonzalez RR, Goforth MH (2007). Macrophage-specific PPAR*γ* controls alternative activation and improves insulin resistance. *Nature*.

[33] Satoh-Asahara N, Shimatsu A, Sasaki Y (2012). Highly purified eicosapentaenoic acid increases interleukin-10 levels of peripheral blood monocytes in obese patients with dyslipidemia. *Diabetes Care*.

[34] Esposito K, Pontillo A, Giugliano F (2003). Association of low interleukin-10 levels with the metabolic syndrome in obese women. *Journal of Clinical Endocrinology and Metabolism*.

[35] Carvalho GQ, Pereira PF, Serrano HMS (2010). Peripheral expression of inflammatory markers in overweight female adolescents and eutrophic female adolescents with a high percentage of body fat. *Applied Physiology, Nutrition and Metabolism*.

[36] Lago R, Gómez R, Lago F, Gómez-Reino J, Gualillo O (2008). Leptin beyond body weight regulation-current concepts concerning its role in immune function and inflammation. *Cellular Immunology*.

[37] Lee YH, Nair S, Rousseau E (2005). Microarray profiling of isolated abdominal subcutaneous adipocytes from obese vs non-obese Pima Indians: increased expression of inflammation-related genes. *Diabetologia*.

[38] Hermsdorff HHM, Puchau B, Zulet MA, Martínez JA (2010). Association of body fat distribution with proinflammatory gene expression in peripheral blood mononuclear cells from young adult subjects. *OMICS*.

[39] Kliewer SA, Sundseth SS, Jones SA (1997). Fatty acids and eicosanoids regulate gene expression through direct interactions with peroxisome proliferator-activated receptors *α* and *γ*. *Proceedings of the National Academy of Sciences of the United States of America*.

[40] Georgiadi A, Kersten S (2012). Mechanisms of gene regulation by fatty acids. *Advances in Nutrition*.

[41] Osborne TF (2000). Sterol regulatory element-binding proteins (SREBPS): key regulators of nutritional homeostasis and insulin action. *Journal of Biological Chemistry*.

[42] Gao L, Wang J, Sekhar KR (2007). Novel n-3 fatty acid oxidation products activate Nrf2 by destabilizing the association between Keap1 and Cullin3. *Journal of Biological Chemistry*.

[43] Francis H, Stevenson R (2013). The longer-term impacts of Western diet on human cognition and the brain. *Appetite*.

[44] Willcox DC, Willcox BJ, Todoriki H, Suzuki M (2009). The Okinawan diet: health implications of a low-calorie, nutrient-dense, antioxidant-rich dietary pattern low in glycemic load. *Journal of the American College of Nutrition*.

[45] Tilley SL, Coffman TM, Koller BH (2001). Mixed messages: modulation of inflammation and immune responses by prostaglandins and thromboxanes. *Journal of Clinical Investigation*.

[46] Miles EA, Allen E, Calder PC (2002). In vitro effects of eicosanoids derived from different 20-carbon fatty acids on production of monocyte-derived cytokines in human whole blood cultures. *Cytokine*.

[47] Samad TA, Moore KA, Sapirstein A (2001). Interleukin-1 *β*-mediated induction of Cox-2 in the CNS contributes to inflammatory pain hypersensitivity. *Nature*.

[48] Lee K-M, Kang B-S, Lee H-L (2004). Spinal NF-kB activation induces COX-2 upregulation and contributes to inflammatory pain hypersensitivity. *European Journal of Neuroscience*.

[49] Greenhough A, Smartt HJM, Moore AE (2009). The COX-2/PGE2 pathway: key roles in the hallmarks of cancer and adaptation to the tumour microenvironment. *Carcinogenesis*.

[50] Hughes-Fulford M (2001). Fatty acid regulates gene expression and growth of human prostate cancer PC-3 cells. *Carcinogenesis*.

[51] Hughes-Fulford M, Li C-F, Boonyaratanakornkit J, Sayyah S (2006). Arachidonic acid activates phosphatidylinositol 3-kinase signaling and induces gene expression in prostate cancer. *Cancer Research*.

[52] Rioux V, Legrand P (2007). Saturated fatty acids: simple molecular structures with complex cellular functions. *Current Opinion in Clinical Nutrition and Metabolic Care*.

[53] Staiger K, Staiger H, Weigert C, Haas C, Häring H-U, Kellerer M (2006). Saturated, but not unsaturated, fatty acids induce apoptosis of human coronary artery endothelial cells via nuclear factor-*κ*B activation. *Diabetes*.

[54] Harvey KA, Walker CL, Pavlina TM, Xu Z, Zaloga GP, Siddiqui RA (2010). Long-chain saturated fatty acids induce pro-inflammatory responses and impact endothelial cell growth. *Clinical Nutrition*.

[55] Murumalla RK, Gunasekaran MK (2012). Fatty acids do not pay the toll: effect of SFA and PUFA on human adipose tissue and mature adipocytes inflammation. *Lipids in Health and Disease*.

[56] Huang S, Rutkowsky JM, Snodgrass RG (2012). Saturated fatty acids activate TLR-mediated proinflammatory signaling pathways. *Journal of Lipid Research*.

[57] Hoenselaar R (2012). Saturated fat and cardiovascular disease: the discrepancy between the scientific literature and dietary advice. *Nutrition*.

[58] Takeuchi H, Sekine S, Kojima K, Aoyama T (2008). The application of medium-chain fatty acids: edible oil with a suppressing effect on body fat accumulation. *Asia Pacific Journal of Clinical Nutrition*.

[59] Nagao K, Yanagita T (2010). Medium-chain fatty acids: functional lipids for the prevention and treatment of the metabolic syndrome. *Pharmacological Research*.

[60] van Dijk SJ, Feskens EJM, Bos MB (2009). A saturated fatty acid-rich diet induces an obesity-linked proinflammatory gene expression profile in adipose tissue of subjects at risk of metabolic syndrome. *American Journal of Clinical Nutrition*.

[61] Dandona P, Aljada A, Bandyopadhyay A (2004). Inflammation: the link between insulin resistance, obesity and diabetes. *Trends in Immunology*.

[62] Brownlee M (2001). Biochemistry and molecular cell biology of diabetic complications. *Nature*.

[63] Marfella R, Esposito K, Giunta R (2000). Circulating adhesion molecules in humans: role of hyperglycemia and hyperinsalinemia. *Circulation*.

[64] Benetou V, Trichopoulou A, Orfanos P (2008). Conformity to traditional Mediterranean diet and cancer incidence: the Greek EPIC cohort. *British Journal of Cancer*.

[65] Willcox BJ, Willcox DC, Todoriki H (2007). Caloric restriction, the traditional okinawan diet, and healthy aging: the diet of the world’s longest-lived people and its potential impact on morbidity and life span. *Annals of the New York Academy of Sciences*.

[66] Brattbakk H-R, Arbo I, Aagaard S (2013). Balanced caloric macronutrient composition downregulates immunological gene expression in human blood cells-adipose tissue diverges. *OMICS*.

[67] Capel F, Viguerie N, Vega N (2008). Contribution of energy restriction and macronutrient composition to changes in adipose tissue gene expression during dietary weight-loss programs in obese women. *Journal of Clinical Endocrinology and Metabolism*.

[68] Dankel SN, Fadnes DJ, Stavrum A-K (2010). Switch from stress response to homeobox transcription factors in adipose tissue after profound fat loss. *Plos One*.

[69] Simopoulos AP (2002). The importance of the ratio of omega-6/omega-3 essential fatty acids. *Biomedicine and Pharmacotherapy*.

[70] HM S (1956). Deficiency of essential fatty acids and atherosclerosis, etcetera. *The Lancet*.

[71] Kromhout D, Yasuda S, Geleijnse JM, Shimokawa H (2012). Fish oil and omega-3 fatty acids in cardiovascular disease: do they really work?. *European Heart Journal*.

[72] Weaver KL, Ivester P, Seeds M, Case LD, Arm JP, Chilton FH (2009). Effect of dietary fatty acids on inflammatory gene expression in healthy humans. *Journal of Biological Chemistry*.

[73] Duda MK, O’Shea KM, Stanley WC (2009). *ω*-3 polyunsaturated fatty acid supplementation for the treatment of heart failure: mechanisms and clinical potential. *Cardiovascular Research*.

[74] Fortin PR, Lew RA, Liang MH (1995). Validation of a meta-analysis: the effects of fish oil in rheumatoid arthritis. *Journal of Clinical Epidemiology*.

[75] Hirsch E, Katanaev VL, Garlanda C (2000). Central role for G protein-coupled phosphoinositide 3-kinase *γ* in inflammation. *Science*.

[76] Rommel C, Camps M, Ji H (2007). PI3K*δ* and PI3K*γ*: partners in crime in inflammation in rheumatoid arthritis and beyond?. *Nature Reviews Immunology*.

[77] Martínez-González MÁ, de la Fuente-Arrillaga C, Nunez-Cordoba JM (2008). Adherence to Mediterranean diet and risk of developing diabetes: prospective cohort study. *BMJ*.

[78] Estruch R, Ros E, Salas-Salvadó J (2013). Primary prevention of cardiovascular disease with a mediterranean diet. *The New England Journal of Medicine*.

[79] Bogani P, Galli C, Villa M, Visioli F (2007). Postprandial anti-inflammatory and antioxidant effects of extra virgin olive oil. *Atherosclerosis*.

[80] Bellido C, López-Miranda J, Blanco-Colio LM (2004). Butter and walnuts, but not olive oil, elicit postprandial activation of nuclear transcription factor *κ*B in peripheral blood mononuclear cells from healthy men. *American Journal of Clinical Nutrition*.

[81] Camargo A, Ruano J, Fernandez JM (2010). Gene expression changes in mononuclear cells in patients with metabolic syndrome after acute intake of phenol-rich virgin olive oil. *BMC Genomics*.

[82] Esposito K, Nappo F, Giugliano F, Giugliano G, Marfella R, Giugliano D (2003). Effect of dietary antioxidants on postprandial endothelial dysfunction induced by a high-fat meal in healthy subjects. *American Journal of Clinical Nutrition*.

[83] Hlebowicz J, Darwiche G, Björgell O, Almér L-O (2007). Effect of cinnamon on postprandial blood glucose, gastric emptying, and satiety in healthy subjects. *American Journal of Clinical Nutrition*.

[84] Qin B, Dawson H, Polansky MM, Anderson RA (2009). Cinnamon extract attenuates TNF-alpha-induced intestinal lipoprotein ApoB48 overproduction by regulating inflammatory, insulin, and lipoprotein pathways in enterocytes. *Hormone and Metabolic Research*.

[85] Markey O, McClean CM, Medlow P (2011). Effect of cinnamon on gastric emptying, arterial stiffness, postprandial lipemia, glycemia, and appetite responses to high-fat breakfast. *Cardiovascular Diabetology*.

[86] Hermsdorff HHM, Zulet MA, Puchau B, Martínez JA (2010). Fruit and vegetable consumption and proinflammatory gene expression from peripheral blood mononuclear cells in young adults: a translational study. *Nutrition and Metabolism*.

[87] Turnbaugh PJ, Ley RE, Mahowald MA, Magrini V, Mardis ER, Gordon JI (2006). An obesity-associated gut microbiome with increased capacity for energy harvest. *Nature*.

[88] Vucenik I, Stains JP (2012). Obesity and cancer risk: evidence, mechanisms, and recommendations. *Annals of the New York Academy of Sciences*.

